# Safety and Early Return to Sports for Early ACL Reconstruction in Young Athletes: A Retrospective Study

**DOI:** 10.3390/medicina60081229

**Published:** 2024-07-29

**Authors:** Yuki Yamanashi, Hirotaka Mutsuzaki, Tatsuhiro Kawashima, Kotaro Ikeda, Masataka Deie, Tomonori Kinugasa

**Affiliations:** 1 Department of Orthopaedic Surgery, Aichi Medical University Hospital, Nagakute 480-1195, Aichi, Japan; 2 Center for Medical Science, Ibaraki Prefectural University of Health Sciences, Ami 300-0394, Ibaraki, Japan; 3 Department of Orthopaedic Surgery, Ibaraki Prefectural University of Health Sciences Hospital, Ami 300-0331, Ibaraki, Japan; 4 Department of Rehabilitation, Ichihara Hospital, Tsukuba 300-3295, Ibaraki, Japan; 5 Department of Orthopaedic Surgery, Ichihara Hospital, Tsukuba 300-3295, Ibaraki, Japan; 6 Department of Orthopaedic Surgery, Hiroshima City Hiroshima Citizens Hospital, Hiroshima 730-8518, Hiroshima, Japan

**Keywords:** early reconstruction, young athletes, anatomic single-bundle reconstruction, outside-in tunnel technique, hamstring autograft

## Abstract

*Background and Objectives*: Although previous reports have shown that early anterior cruciate ligament (ACL) reconstruction is associated with an increased risk of stiffness, recommendations for delayed surgery are based on outdated literature. The advent of arthroscopic surgery and accelerated rehabilitation protocols warrants a reexamination of the optimal surgical timing. The purpose of this study was to investigate complications during early ACL reconstruction after injury in young athletes. *Materials and Methods*: A total of 87 patients (27 males and 60 females) were included in this study. Patients who underwent anatomic ACL reconstruction using hamstring autografts were evaluated. Patients under 25 years of age with a Tegner activity score greater than 6 were included and classified into three groups according to the time from injury to surgical treatment: one week from injury to surgery (early group), three to six weeks from injury to surgery (normal group), and three to six months from injury to surgery (delayed group). We evaluated the rates of various complications such as graft rupture, contralateral injury, the need for manipulation for loss of ROM, infection, and fracture around the knee up to 2 years postoperatively. In addition, we investigated postoperative muscle strength, Lysholm score, Tegner activity score, and period of the return to sport from injury. *Results:* Patients in the delayed group were younger than those in the other groups (*p* = 0.009). Patients in the early group had a lower range of motion than those in the other groups preoperationly. However, the 1-month postoperative range of motion was comparable between groups. Patients in the early group had greater postoperative Tegner activity scores than those in the other groups. The period of return to sport from injury in the delayed group was longer than in the other groups. There were no statistically significant differences in the postoperative complication rate, muscle strength, or Lysholm score. *Conclusions*: ACL reconstruction performed 1 week from injury to surgery in young athletic patients indicated the rate of complications were not significantly different among the groups. Early ACL reconstruction with no postoperative complications may be related to early return to sports and a high level of sports.

## 1. Introduction

There has been considerable debate regarding the optimal timing for anterior cruciate ligament (ACL) reconstruction. Shelbourne et al. [1] reported that delaying reconstruction at least three weeks after injury resulted in a significant decrease in the incidence of arthrofibrosis and a decrease in the lack of full extension. Moreover, several studies have reported that early reconstruction is associated with an increased risk of stiffness [2,3]. A loss of ≥ 5 degrees of extension has been reported to cause an abnormal gait that can lead to patellofemoral pain and quadriceps weakness [4]. Although a delay in surgical reconstruction has been recommended for achieving a full range of motion (ROM), recommendations to delay surgery have been based on studies from over 20 years ago with outdated surgical techniques and rehabilitation. Furthermore, ACL reconstructions in these studies were performed without the use of arthroscopy and with the use of more restrictive rehabilitation protocols [1,2,3]. Bottoni et al. reported that early ACL reconstruction using arthroscopy, early mobilization, and maintenance of extension did not result in loss of motion or suboptimal clinical results [5]. Several recent studies have reported no functional disadvantages between early and delayed ACL reconstruction [6,7,8,9,10,11]. However, the definition of early surgery was not standardized in these reports, and no clear evidence was provided to determine the optimal timing of surgery.

The early return to sport is highly important for young athletes. Several studies have shown that early surgical treatment is associated with superior postoperative muscle strength and Lysholm scores [9,10,12]. However, the superiority of early reconstruction for improved functional recovery remains insufficient. If early reconstruction is safe and provides clinical benefits, we can proactively recommend this operation. To perform this operation, the outcomes and complication risks between early and delayed reconstructions need to be evaluated and compared.

The purpose of this study was to evaluate various complications (graft rupture, contralateral injury, need for manipulation for loss of ROM, infection, and fracture around the knee after surgery) to assess the safety of early ACL reconstruction compared to other surgical timing groups. We divided patients into three groups (1 week, 3 to 6 weeks, and 3 to 6 months from injury to surgery), with a particular focus on the early period (one week from injury to surgery), while investigating the optimal timing of surgery, as highlighted in a previously reported study [12]. Furthermore, we investigated postoperative muscle strength, Lysholm score, Tegner activity score, and period of return to sport after injury. We hypothesized that early surgery does not increase the risk of complications and is related to early recovery of muscle strength and a return to the same level of sports prior to injury. Furthermore, patients who underwent early reconstruction could potentially return to sports earlier due to a lack of waiting time for the operation.

## 2. Materials and Methods

This retrospective study was reviewed and approved by the ethics committee of Ichihara Hospital (approval number 1901). We obtained patient consent by opt-out. A total of 592 patients who underwent primary ACL reconstruction between April 2012 and January 2020 at our hospital were assessed for eligibility for the study. Patients who underwent ACL reconstruction up to six months after injury, anatomic single-bundle ACL reconstruction using hamstring autografts and the outside-in technique, and follow-up examination for a minimum of 24 months were included. A previous study showed that athletes younger than 25 years who returned to sports after ACL reconstruction had a high secondary ACL injury rate [13]. Thus, we included patients under 25 years of age with a Tegner activity score of more than 6.

**Figure 1:**
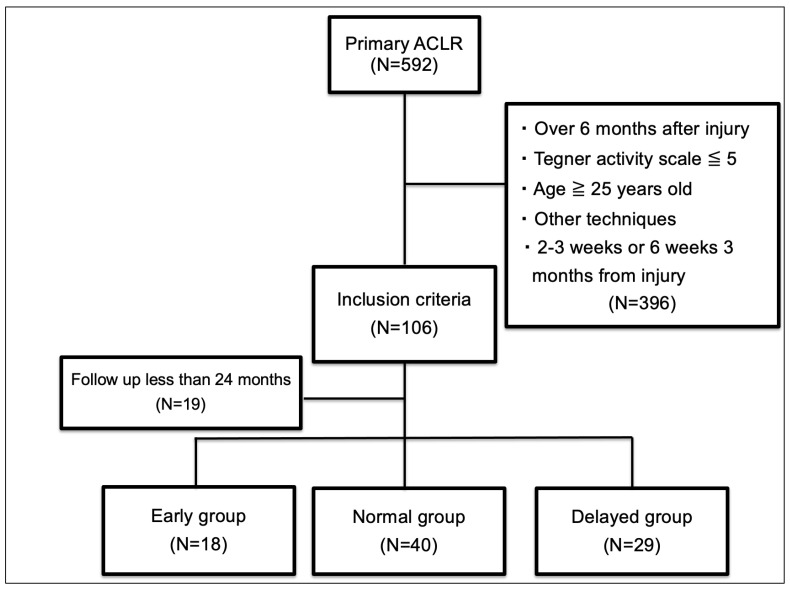
Flow chart of patient inclusion. ACLR, anterior cruciate ligament reconstruction.

Patients were excluded if they had undergone ACL reconstruction more than six months after injury, ACL reconstruction using other operative techniques (double bundle, transtibial tunnel approach), ligament reconstruction, quadriceps tendon graft or bone patella tendon bone graft, revision ACL reconstruction, or bilateral ACL reconstruction. Moreover, patients who underwent surgery between each period were excluded (Figure 1). ACL reconstructions were performed by two experienced surgeons.

The definitions of “early” and “delayed” ACL reconstructions have varied widely in previous studies. A recent systematic review reported that the definition of “early” ranged from 8 to 10 days from injury to surgery, and “delayed” ranged from 4 weeks to greater than 3 months [12]. We focused on the first week post-injury and, referencing Shelbourne’s study, used cases beyond three weeks as the primary control. In accordance with our country’s guidelines, we conducted the study using three to six months post-injury as the secondary control [1]. To address this terminological inconsistency in the literature, we stratified patients who underwent ACL reconstruction into three groups based on the time from injury to surgical treatment: one week from injury to surgery (early group), three to six weeks from injury to surgery (normal group), and three to six months from injury to surgery (delayed group).

The timing of the surgery should be performed as soon as possible to prevent subsequent meniscal or cartilage damage and to achieve an early return to sports. However, the timing of the consultation for injuries depended on the patient. Similarly, some patients had personal circumstances, such as schoolwork and jobs. Therefore, these factors influenced the timing of the operation.

The surgical procedure used in this study was based on an arthroscopic anatomical single-bundle ACL reconstruction previously described by Yamanashi et al. [14] The graft was made of the semitendinous tendon alone or both the semitendinous and gracilis tendons. After the tendons were harvested, an arthroscopic evaluation was performed on all patients to assess ACL, meniscus, and cartilage injuries. After routine arthroscopy, patients with meniscus tears underwent partial meniscectomy or meniscal repair, while patients with cartilage damage underwent drilling. For ACL reconstruction, anatomical tunnels were created via the outside-in technique, and each bone tunnel was positioned at the center of the footprints. After creating a multi-strand tendon graft, the femoral end of the graft was passed through a TightRope RT (Arthrex, Naples, FL, USA), and the tibial end of the graft was sutured using a FiberLoop or TigerLoop (Arthrex, Naples, FL, USA). Finally, the graft was passed through both tunnels and fixed at 20° knee flexion, and mild tension was applied using a Double Spiked Plate (Smith and Nephew, Andover, MA, USA) on the tibial side.

The postoperative rehabilitation protocol was as follows. The patients started partial weight-bearing and ROM exercise at 2 days and full weight-bearing walking at 3 weeks after surgery. Patients who underwent meniscal repair began ROM exercise 2 weeks after surgery. If patients passed the isokinetic test with greater than 70% limb symmetry, they were allowed to start running 3 months postoperatively. If patients passed the isokinetic test with greater than 80% limb symmetry, they were allowed to start jumping and perform agility training 6 months postoperatively. Finally, if patients were able to stand up from a 10-cm step, pass a single hop test with greater than 90% limb symmetry, and improve their movement in each sport, they were allowed to return to sport at 9–12 months after surgery. The postoperative rehabilitation schedule was the same for each group.

Patient data were collected from the clinical and operation records of our hospital. Complications were investigated, including graft rupture, contralateral ACL injury, need for manipulation for loss of ROM, infection, and fracture around the knee up to 2 years postoperatively. Graft ruptures were defined as patients who underwent revision ACL reconstruction or had a second injury episode with grafts classified as International Knee Documentation Committee (IKDC) [15] grade C or D. The primary outcome was to evaluate the rate of each complication.

Furthermore, the characteristics of patients (preoperative age, height, weight, sex, Tegner activity score, and presence of meniscus tear) were compared to those of patients with secondary outcomes, including postoperative ROM, knee laxity, muscle strength, Lysholm score, Tegner activity scale score, and the period of return to sports from injury. The return to sports was defined as participating in all practices and the form of games. We only compared patients who had no complications during the evaluation of postoperative function in each group. The ROM was measured four times (preoperatively and at 1 month, 3 months, and 12 months postoperatively). Knee laxity was assessed with a KT-1000 arthrometer (12 months postoperatively). Muscle strength, isokinetic concentric quadriceps strength, and hamstring strength were measured two times (3 months and 6 months postoperatively) using a Biodex system 3 (SAKAI MED, Tokyo, Japan) at 60°/s.

Statistical analysis was performed using IBM SPSS Statistics version 29 (IBM Corp., Armonk, NY, USA). Patient age, height, weight, Lysholm score, Tegner activity score, range of motion, and muscle strength among the three groups were evaluated using one-way ANOVA. When used as a post hoc test, the Bonferroni test was performed. Patient sex, knee laxity, meniscus tears, and complications were evaluated using the χ^2^ test. A *p* value of <0.05 was considered statistically significant.

A power calculation was performed using the G*Power3 procedure with a confidence level of 95% (α = 0.05), a power (1–β) of 80%, and an effect size of 0.40, resulting in an estimated sample size of 66 patients. 

## 3. Results

### 3.1. Patient Characteristics

The descriptive data for the 87 patients included in this study are summarized in Table 1. Statistical analysis revealed a significant difference between each group in terms of patient age and mean time from injury to operation (*p* < 0.001, respectively). The delayed group was younger than the other groups (early group vs. delayed group *p* = 0.047; normal group vs. delayed group *p* = 0.014). Height, weight, sex, preoperative Tegner activity level, and rate of concomitant meniscal tear were not significantly different among the three groups.

### 3.2. Postoperative Complications

**Figure 2:**
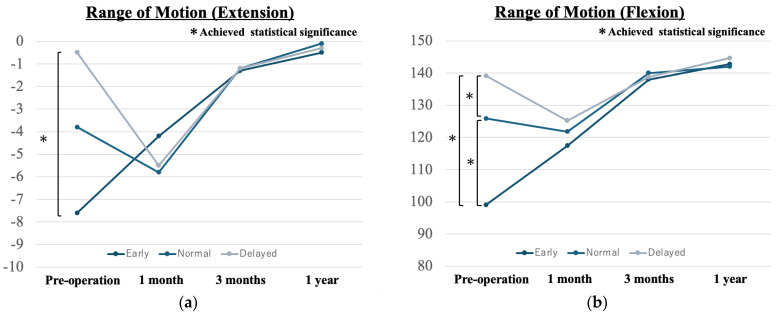
(**a**,**b**) Pre- and Postoperative ROM.

Patients in the early group had a lower preoperative ROM than did those in the other groups (*p* = 0.015, *p* < 0.001, and *p* = 0.017, respectively). However, postoperative ROM after 1 month was not significantly different among the three groups. The average preoperative knee flexion angle was 100 ± 24 degrees in the early group, 125 ± 17 degrees in the normal group, and 140 ± 9 degrees in the delayed group. There were significant differences among the three groups (*p* = 0.015, *p* < 0.001, and *p* = 0.017, respectively). However, the postoperative knee flexion angle after 1 month was not significantly different among the three groups. The average preoperative knee extension angle was −8 ± 10 degrees in the early group, −4 ± 7 degrees in the normal group, and 0 ± 5 degrees in the delayed group. The early group had a statistically lower extension range than the normal group. On the other hand, the postoperative range at 1 month was comparable among the three groups (Figure 2). Additionally, the number of manipulations for loss of ROM was 1 (1/18 = 5.6%) in the early group, 1 (1/40 = 2.5%) in the normal group, and 0 (0/29 = 0%) in the delayed group. There were no significant differences among the three groups (*p* = 0.463). The rates of other complications, such as graft rupture, contralateral ACL injury, infection, fracture, and stability test according to measurements on the KT-1000 arthrometer, were comparable in all three groups (Table 2).

### 3.3. Postoperative Sports Activity

The mean 1-year postoperative Tegner activity scores were 7.9±1.7 in the early group vs. 6.9±1.0 in the normal group and vs. 6.7 ± 1.0 in the delayed group. Patients who had no complications in the early group had greater Tegner activity scores than those in the other groups (early group vs. delayed group *p* = 0.029, early group vs. delayed group *p* = 0.012). The mean periods of return to sports from injury were 8.6 ± 1.6 months in the early group vs. 10.2 ± 1.9 months in the normal group and vs. 14.1 ± 2.8 months in the late group. The delayed group was longer than the other groups (early group vs. delayed group *p* < 0.001, normal group vs. delayed group *p* < 0.001). Muscle strength at 3 and 6 months after surgery and the Lysholm score at 1 year after surgery were comparable in all three groups (Table 3).

## 4. Discussion

In this study, the risk of early ACL reconstruction was investigated in young athletic patients. The results of the ACL reconstruction within 1 week of injury indicated that the rate of various complications did not increase in comparison with those within 3 to 6 weeks and 3 to 6 months of injury. In addition, early ACL reconstruction may be associated with an early return to sports. Moreover, muscle strength at 3 and 6 months and the Lysholm score were not greater in the early group than in the other groups.

Our study has several strengths. First, only young athletic patients were included. We believe that the indications for early ACL reconstruction primarily apply to young athletes. Therefore, in this study, we focused our evaluations on young athletes. Second, we compared three periods in terms of surgical timing. Previous studies have limited their comparisons to early and delayed operations. Furthermore, definitions of early and delayed operations have varied between studies. By comparing the three timing periods, we were able to make a more detailed assessment.

In this study, we mainly investigated various complications of early ACL reconstruction. In particular, loss of ROM and graft failure caused poor outcomes after surgery. A safe return to sports is the most important consideration in ACL reconstruction; therefore, we focused on complications that may affect this goal. The most concerning complication in early surgery is joint stiffness, and several studies have reported that early reconstruction is associated with an increased risk of stiffness [1,2,3]. Limited preoperative ROM and perioperative pain are conducive to arthrofibrosis [16]. In our study, preoperative ROM was limited in the early group. Thus, residual limitations in postoperative ROM were of concern. However, our results showed that postoperative ROM was comparable in all three groups after 1 month. Meighan et al. [17] reported no significant difference in the loss of ROM after 6 weeks postoperatively between early and delayed surgery. Our study revealed similar postoperative ROMs between the groups. Moreover, a previous study showed that the incidence of joint stiffness after ACL reconstruction was between 4% and 38% [18]. In early intervention, only one patient ultimately required surgery due to a loss of ROM. Similar to previous reports, our results suggest that early surgery using arthroscopy and accelerated rehabilitation with early knee movement are important for preventing joint stiffness.

The present study showed that the rate of graft rupture was comparable in all three groups. The rate of graft rupture in the early group was 11.1%. Previous studies reported that the failure rate of single-bundle reconstruction ranged from 3.2 to 15% [19,20]. Although our failure rate was slightly high, a previous review showed that athletes younger than 25 years who returned to sports after ACL reconstruction had a high secondary ACL injury rate [13]. Grindem et al. showed that the knee reinjury rate was more than 4 times greater in ACL-reconstructed patients who returned to jumping, pivoting, and hard cutting sports [21]. In this study, we included only patients younger than 25 years who hoped to return to sports at a high level of performance. Therefore, the patients in this study had a greater risk of re-injury. Although it is essential to pay sufficient attention to prevent reinjury, our results indicate that early surgery might not increase the rate of graft rupture.

The patients in the early group had a higher Tegner activity score and earlier return to sports from injury than those in the other groups. On the other hand, there were no significant differences in muscle strength in this study. Several studies have shown that preoperative muscle strength influences muscle recovery and subject scores after ACL reconstruction [22,23,24]. Thus, we hypothesized that early operations performed before the development of muscle atrophy are related to early recovery of muscle strength. However, postoperative muscle strengths were comparable among the three groups. In all patients who develop muscle atrophy, impairment may linger for 6 months after surgical treatment [25]. In addition, a previous study showed that age is a predictor of residual muscle atrophy [26]. We only included young athletes in this study, and they were highly motivated to return to sports. For this reason, we expected that early ACL reconstruction would lead to the early restoration of postoperative muscle strength. Although the results obtained in the present study were not what we expected, early operations showed a reduction in recovery time that was proportional to the reduction in delay to surgery. According to these results, we may be able to explain why muscle strength will be of comparable weakness, even if early ACL reconstruction is chosen.

Early ACL reconstruction was associated with concomitant meniscal tears. Delaying surgical reconstruction can potentially lead to subsequent episodes of instability and may result in concomitant knee pathologies [27]. Dysfunction of the ACL leads to subsequent meniscal and osteochondral lesions. Some papers have indicated an increased incidence of meniscal tears in individuals who underwent ACL reconstruction more than 6 to 12 months after injury [27,28]. Therefore, early intervention might reduce the risk of secondary meniscal tears. In this study, all patients underwent surgical treatment within 6 months after injury. Therefore, we considered that the risk of second injuries was not high, even in the delayed group. Future advantages in early reconstruction may be found compared with reconstruction more than 6 months after injury. On the other hand, the meniscus has healing potential, and previous studies have shown that untreated meniscus tears with ACL reconstruction do not require reoperation, especially small and peripheral lateral tears [29,30]. Therefore, we need to keep in mind the possibility of treating meniscal tears that can heal naturally.

There were several limitations to this study. First, this was a non-randomized retrospective study. Some data were missing, especially in terms of muscle strength and stability tests. Missing data might influence our results to some extent. Second, the timing of the return to sport and the definition of return to sport were variable. Although we generally allowed patients to return to sports after they passed each test, some patients returned to sports before passing each test due to special circumstances, such as the need to return to sports to obtain scholarships or participate in an important game. Third, the sample size of this study was small and unequal. Fourth, we did not evaluate the classification of the original ACL injury, cartilage damage, or osteoarthritis after the operation. These factors might affect clinical outcomes. Further studies should be conducted with a greater and equal number of patients.

## 5. Conclusions

ACL reconstruction performed within 1 week of injury in young athletic patients indicated that the rates of various complications were not significantly different among the groups. Early ACL reconstruction with no postoperative complications may be related to an early return to sports and a high level of sports.

## Figures and Tables

**Table 1:** Patient Characteristics.

Characteristics	Early Group (N = 18)	Normal Group (N = 40)	Delayed Group (N = 29)	*p* Value	Effect Size	Power
Mean time; injury to operation (day)	4.2 ± 1.5 (2–7)	32.7 ± 5.8 (22–42)	122.2 ± 25.8 (90–179)	<0.01 *	0.93	1.0
Age (year)	19.9 ± 3.0 (14–25)	19.3 ± 3.0 (15–25)	17.2 ± 2.8 (14–24)	<0.01 *	0.23	0.8
Height (cm)	163.1 ± 9.1 (152–183)	164.1 ± 8.3 (151–182)	163.3 ± 8.2 (152–180)	0.88	0.04	0.07
Weight (kg)	61.6 ± 10.3 (51–84)	62.4 ± 14.2 (46–106)	61.6 ± 12.1 (41–85)	0.96	0.14	0.57
Male	7	11	9	0.69	0.09	0.11
Female	11	29	20			
Mean TAS	7.4 ± 1.2	7.1 ± 1.0	6.8 ± 0.6	0.09	0.16	0.48
6	6	12	9			
7	4	20	19			
8	2	1	0			
9	4	7	1			
10	2	0	0			
Total meniscus tear	11	20	15	0.73	0.09	0.73
MM tear	1	2	1	0.93		
LM tear	7	16	11	0.98		
MM + LM tear	3	2	3	0.35		
Concomitant meniscal suture	10	17	15	0.59	0.11	0.14

Mean time, age, height, weight, and mean TAS are presented as mean ± SD. We only included patients who were under 25 years old with a Tegner activity score over 6. * Achieved statistical significance between each group. TAS: Tegner activity score, MM: medial meniscus, LM: lateral meniscus.

**Table 2:** Complications and Stability Test.

	Early Group N = 18	Normal Group N = 40	Delayed Group N = 29	*p* Value	Effect Size	Power
Total complications	6 (33.3%)	9 (22.5%)	6 (20.7%)		0.11	0.14
Graft rupture	2 (11.1%)	5 (12.5%)	2 (6.9%)	0.75		
Contralateral injury	2 (11.1%)	2 (5%)	3(10.3%)	0.63		
Meniscal tear	1 (5.6%)	1 (2.5%)	1 (3.4%)	0.84		
Manipulation	1 (5.6%)	1 (2.5%)	0 (0%)	0.46		
Infection	0 (0%)	0 (0%)	0 (0%)			
Fracture	0 (0%)	0 (0%)	0 (0%)			
KT-1000	N = 11	N = 30	N = 18	0.42	0.42	0.15
≤−2 mm	0% (0/11)	3.3% (1/30)	5.6% (1/18)			
−1~2 mm	100% (11/11)	83.3% (25/30)	83.3% (15/18)			
≥3 mm	0% (0/11)	13.3% (4/30)	11.1% (2/18)			

**Table 3:** Isokinetic Concentric Muscle Strength and Tegner Activity Score.

	Early Group	Normal Group	Delayed Group	*p* Value	Effect Size	Power
3 months	N = 14	N = 30	N = 22			
Hamstrings (%)	69.5 ± 17.2	74.6 ± 14.7	79.6 ± 14.0	0.15	0.18	0.39
Quadriceps (%)	69.2 ± 15.5	73.9 ± 19.2	73.5 ± 12.3	0.66	0.09	0.12
6 months	N = 15	N = 20	N = 20			
Hamstrings (%)	87.5 ± 16.9	88.2 ± 19.4	87.3 ± 10.1	0.98	0	0.05
Quadriceps (%)	81.2 ± 16.2	80.6 ± 16.6	82.1 ± 10.1	0.95	0.03	0.06
1 year	N = 12	N = 30	N = 20			
Lysholm score	98.3 ± 2.5	98.9 ± 2.4	98.2 ± 4.9	0.80	0.01	0.08
Mean TAS	7.9 ± 1.7	6.9 ± 1.0	6.7 ± 1.0	0.01 *	0.15	0.81
4	1	0	1			
5	0	1	1			
6	1	6	5			
7	3	18	14			
8	2	1	0			
9	3	3	1			
10	2	0	0			
Time to return to sport from injury (months)	8.6 ± 1.6	10.2 ± 1.9	14.1 ± 2.8	<0.01 *	0.5	1

Muscle strength, Lysholm score, mean TAS, and time to return to sport from injury are presented as mean ± SD. * Achieved statistical significance. TAS: Tegner Activity Score.

## Data Availability

The datasets generated and/or analyzed during the current study are available from the corresponding authors upon reasonable request.
